# Perceptual Compensation Is Correlated with Individuals' “Autistic” Traits: Implications for Models of Sound Change

**DOI:** 10.1371/journal.pone.0011950

**Published:** 2010-08-19

**Authors:** Alan C. L. Yu

**Affiliations:** Phonology Laboratory, Department of Linguistics, University of Chicago, Chicago, Illinois, United States of America; University of Minnesota, United States of America

## Abstract

Variation is a ubiquitous feature of speech. Listeners must take into account context-induced variation to recover the interlocutor's intended message. When listeners fail to normalize for context-induced variation properly, deviant percepts become seeds for new perceptual and production norms. In question is how deviant percepts accumulate in a systematic fashion to give rise to sound change (i.e., new pronunciation norms) within a given speech community. The present study investigated subjects' classification of /s/ and /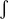/ before /a/ or /u/ spoken by a male or a female voice. Building on modern cognitive theories of autism-spectrum condition, which see variation in autism-spectrum condition in terms of individual differences in cognitive processing style, we established a significant correlation between individuals' normalization for phonetic context (i.e., whether the following vowel is /a/ or /u/) and talker voice variation (i.e., whether the talker is male or female) in speech and their “autistic” traits, as measured by the Autism Spectrum Quotient (AQ). In particular, our mixed-effect logistic regression models show that women with low AQ (i.e., the least “autistic”) do not normalize for phonetic coarticulation as much as men and high AQ women. This study provides first direct evidence that variability in human's ability to compensate for context-induced variations in speech perceptually is governed by the individual's sex and cognitive processing style. These findings lend support to the hypothesis that the systematic infusion of new linguistic variants (i.e., the deviant percepts) originate from a sub-segment of the speech community that consistently under-compensates for contextual variation in speech.

## Introduction

A ubiquitous feature of speech is its great variability depending on its acoustic, phonological, semantic, and syntactic contexts. To understand speech, the listener must take into account context-induced effects to recover the intended message. This type of context-induced adjustment in speech perception is called *perceptual compensation* (PC). Consider, for example, the perception of sibilant sounds such as the initial /s/ in “Sue” and /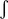/ in “shoe”. Acoustically, /s/ is more /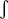/-like next to a rounded vowel such as /u/ due to the noise frequency lowering effect of lip protrusion in natural coarticulated speech. Nonetheless, listeners hear more /s/ when an ambiguous sibilant is followed by /u/ than when it is followed by an unrounded vowel, such as /a/ [1], [2], presumably because listeners take into account the lowered noise frequencies of /s/ in a rounded vowel context. Socio-indexical information may also affect speech perception. Ambiguous sibilants are perceived more often as /s/ when the talker is male than when the talker is female [3], even though /s/ is acoustically more /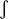/-like when produced by male talkers. This type of compensation for talker voice is evident even when the talker voice is gender-ambiguous; if the listener believes the talker to be male, then she compensates accordingly [4].

Understanding of human's ability to normalize for context-induced variation in speech has serious implication for our understanding of language change. Many theorists have likened language change to biological evolution in having a two-step process of variation and selection [5]–[8]. New variants propagate across a speech community as a result of a process of selection and rejection by language users who evaluate all variations with respect to their social, articulatory, perceptual, and lexical-systematic dimensions. The sources of variation are many [5], [8]–[10]. Setting aside the influence of language contact, one of the primary sources of language-internal variation is hypothesized to have originated from listeners failing to compensate for context-induced variation in speech properly [7], [9], [10]. Errors in perception may lead to adjustments in perceptual and production norms. Thus in the case of sibilants, speakers might mistake /su/ for /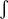
u/ and subsequently start producing /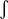
u/. When such new variants come to be associated with social significance and are spread to the rest of the speech community, sound change obtains [11], [12]. An explanatory theory of sound change, and of language change in general, must therefore not only explain the origins of variation, but also take into account the orderly differentiation in a language serving a community [13], as reflected in correlations between linguistic variation and such macrosocial structures as socioeconomic class, ethnicity, and gender [12]. Notwithstanding the complexity of understanding how social significance comes to be associated with certain linguistic features, it remains unclear what mechanism leads to the initial adjustments in the first place. That is, under what circumstances would individuals systematically under-/over-compensate for context-induced variation in speech and create competing perceptual and production norms in the speech community? How are these new norms distributed in the speech community to give rise to the type of orderly differentiations constitutive of social structures? Focusing on gender-differentiated linguistic variation, this study advances the hypothesis that individuals in a speech community vary in their abilities to perceptually normalize for context-induced variation according to differences in their cognitive processing styles. As variability in cognitive processing style, estimated in terms of individual differences in Autism Spectrum Quotient (AQ), is gender-differentiated, variation in perceptual and production norms may also be similarly structured along the gender dimension.

### Cognitive processing styles

Cognitive processing style refers to psychological dimensions representing preferences and consistencies in an individual's particular manner of cognitive functioning, with respect to acquiring and processing information [14]–[16]. Although many people who write about cognitive processes, including philosophers such as Locke and Hume, assume implicitly or explicitly that they are the same for all normal adults, individual differences in cognitive processing styles are evident at all levels of human cognition. Some studies attribute differences in cognitive processing styles to hemispheric specialization, showing that the left hemisphere of the brain tends to have more verbal and sequential abilities; whereas, the right hemisphere tends to excel in processing visual-spatial tasks [17]–[20]. Others attribute variations to socio-cultural differences, arguing that social interdependence fosters holistic cognition (i.e. a tendency to attend to the broad perceptual and cognitive field rather than to a focal object and its properties) and a tendency to reason in terms of relationships and similarities, rather than rules and categories [21]–[23].

Differences in cognitive processing style have also been associated with individuals with different ranges of “autistic” traits. While the diagnosis of an autism spectrum condition (ASC), of which classic autism and Asperger Syndrome are the clearest subgroups, involves difficulties in social development and communication, alongside the presence of unusually strong repetitive behavior or ‘obsessive’ interests [24], [25], many cognitive theories of ASC have in recent years emphasized the importance of taking into account not only the cognitive and social deficits people with ASC exhibit but also aspects of the cognitive abilities that are left intact or even enhanced. The Weak Central Coherence (WCC) theory, for example, argues that children and adults with autism show “detail-focused processing in which features are perceived and retained at the expense of global configuration and contexualized meaning” [26], while individuals with normal central coherence tend to process incoming information by pulling information together for higher-level meaning often at the expense of memory for detail [27]. The Enhanced Perceptual Functioning (EPF) model proposes that superiority of perceptual flow of information in individuals with ASC in comparison to higher-order operations leads to difficulties in controlling perceptual processes, which may in turn be disruptive to the development of other behaviors and abilities [28]. The Empathizing–Systemizing (E–S) Theory of autism attempts to explain delays and deficits in empathy while at the same time accounting for the areas of strength by reference to intact or even superior skill in systemizing [29]. Children with ASC, for example, have been shown to perform above the level that one would expect for their age on a physics test [30]. Those with high-functioning autism (HFA) or Asperger syndrome also score higher on the Systemizing Quotient than people in the general population [31].

Differences in cognitive processing styles associated with individuals with ASC might also have linguistic consequences. Within the domain of language and communication, there is evidence that ASC individuals' “lexical” abilities involving individual words are spared or enhanced (e.g., in picture naming; [32]), even when the “pragmatics” of language use (i.e. the usage of language in social and communicative contexts) is hampered (see [33] for a recent review). At the lower level of speech processing, individuals with ASC have also been found to possess certain enhanced auditory and perceptual abilities. Individuals with high AQ, for example, are found to possess high auditory sensitivity or enhanced perceptual processing [28]. HFA individuals might have enhanced discrimination of pure tones [34]. Finally, HFA individuals exhibit canonical categorical perception in visual categorization, although they do not show a facilitation of discrimination near the boundary between categories, suggesting an increased autonomy of low-level perceptual processes in autism in the form of a reduced top-down influence from categories toward discrimination [35].

Of particular relevance in the present context is the fact that “autistic” traits (or “the broader phenotype”; [36]) are found not only at a high level in people with autism spectrum condition but are also found on a continuum at lower levels throughout the population [31], [37]–[39]. This continuum is revealed using instruments such as the Autism Spectrum Quotient (AQ) [38], the Empathy Quotient (EQ) [39], and the Systemizing Quotient (SQ) [31], which measure such individual differences. Men, for example, generally have higher AQ scores than females; scientists, particularly mathematicians, also tend to outscore social scientists and humanists ([31], [38], [39]; cf. [40]). Such individual differences in “autistic” traits have recently been shown to correlate with individuals' speech processing. Total AQ score taken from within the neurotypical population is found to correlate significantly negatively with the extent of identification shift associated with the ‘Ganong effect’ (i.e. the bias in categorization in the direction of a known word) [41].

In light of these findings, the present study was designed to investigate whether the extent of perceptual compensation varies as a function of cognitive processing style by testing neurotypicals' ability to normalize for talker voice and for coarticulation in speech and correlating the perceptual results with the individual's AQ. As individuals with ASC are characterized by deficits in social cognition and communication, the present study investigated whether talker voice compensation would vary as a function of an individual's ability to incorporate socio-indexical information in speech processing. Despite findings that the perception of gender according to talker voice is not impaired in HFA children, their response time profiles are different from the matched controls [42]. It is also not clear whether individuals with high AQ can integrate talker voice information in perceptual normalization. Perceptual compensation for coarticulation is chosen to test listeners' abilities to integrate syntagmatic contextual information in speech processing. High AQ neurotypicals might show impaired abilities to perceptually compensate for phonetic context-induced variation in speech if the processing of phonetic context information involves the same mechanism that triggers deficits in integrating information into a meaningful whole associated with ASC individuals. On the other hand, individuals with ASC exhibit increased ability to systemize (i.e. creating systematic association between objects or features). This enhanced ability to systemize rule-governed patterns, if extended to the neurotypicals with high degree of “autistic” traits, might increase such individuals' ability to keep track of syntagmatically-governed phonetic variation in speech, which in turns might heighten their ability to compensate for such variation in the signal. Finally, it is worth noting that, from the perspective of sound change research, the Autism Spectrum Quotient (AQ) is a uniquely apt instrument for investigating linkages between individual differences in cognitive processing style and sound-change inducing perceptual compensation variation. To begin with, traits as assessed by the AQ are found to have high heritability [43] as well as to be stable cross-culturally [44]. The AQ has not only been shown to correlate with differences in speech processing [41], a link of AQ with differences in personality traits (e.g., neuroticism, extraversion, agreeableness, and conscientiousness) has also been established [45], [46]. Thus, establishing a relationship between individual AQ variation to differences in speech processing might provide crucial information for understanding the social dynamics that enable socially-motivated sound change to occur within a speech community.

## Results

### Correlation with AQ

Descriptive statistics of the quotient scores are summarized in Table 1. The distributions of AQ scores were typical of normally developing populations. As a general comparison, the mean total AQ of individuals with ASC (N = 58) in Baron-Cohen et al.'s study [38] is 35.8 (SD = 6.5), while the mean total AQ of the Cambridge University students they surveyed (N = 840) is 17.6 (SD = 6.4). Applying Baron-Cohen et al's scoring method (they did not calculate the AQ on a Likert-scale as in the present study), subjects in the present study has a mean total AQ of 18.45 (SD = 8.25).

**Table 1:** Descriptive statistics of measured factors.

Factor	Sex	Mean	Range	SD
Overall AQ		**110.05**	**78–155**	**18.00**
	*f*	108.72	78–155	18.53
	*m*	111.57	80–151	17.59
Social Skills (AQSS)		**19.90**	**12–33**	**5.89**
	*f*	20.25	12–33	5.67
	*m*	19.5	12–31	6.22
Attention Switching (AQAS)		**24.53**	**15–36**	**4.69**
	*f*	24.28	17–35	4.68
	*m*	24.82	15–36	4.78
Attention to detail (AQAD)		**26.87**	**15–37**	**5.20**
	*f*	26.38	15–37	5.12
	*m*	27.43	18–37	5.32
Communication (AQCM)		**19.23**	**10–33**	**5.03**
	*f*	19.06	10–33	5.56
	*m*	19.43	11–27	4.43
Imagination (AQIM)		**19.68**	**10–30**	**4.39**
	*f*	18.75	10–28	4.54
	*m*	20.75	13–30	4.04

Scores averaged across the sexes are bolded. All scales were scored in such a way that a high score is more “autistic”, i.e. lower social skills, difficulty in attention switching, high attention to detail and patterns, lower ability to communicate, low imagination.

**Figure 1:**
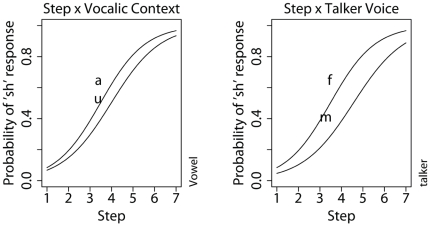
Interaction between continuum step and vocalic context (left panel) and between continuum step and talker voice (right panel). The predictor variables were back-transformed to their original scales in the figure.

**Figure 2:**
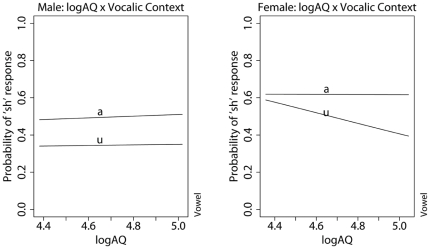
Interaction between vocalic context and subject's log-transformed total AQ score according to subject's sex.

Subject responses were modeled using a linear mixed-effect model with a logistic link function [47]. The model was fitted in *R* [48], using the *lmer*( ) function from the *lme4* package for mixed-effect models [49]. The dependent variable is subject's selection of /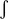/. Subjects' /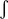/ responses were coded as 1 and /s/ responses as 0; positive regression weights indicate that a high value in a predictor variable makes a /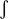/ response more likely. The model contains seven fixed variables: Trial (1–112), Block (1–3), Continuum Step (1–7), Talker.Voice (male vs. female), Vowel (/a/ vs. /u/), Subject.Sex (male vs. female), and total AQ (50–200). The model also includes six two-way interactions: Step x Talker.Voice, Step x Vowel, Talker x Vowel, Vowel x Subject.Sex, Vowel x AQ, and Subject.Sex x AQ and one three-way interaction: Subject.Sex x Vowel x AQ. Additionally, the analysis includes Trial nested within Block as a random factor and two by-subject random slopes, for Trial and for Block. The initial model included the effects of the control variables (Trial, Block, Subject.Age), Subject.Sex, Continuum Step, Talker.Voice, Vowel, total AQ, and interactions (two-way interactions between Continuum Step and Talker.Voice or Vowel, between total AQ and Subject.Sex, Talker.Voice, or Vowel, and three-way interactions between total AQ, Subject.Sex, and Talker.Voice or Vowel) as fixed factors, as well as Trial and Block nested within Subject as random slopes and Trial nested within Block as a random effect. The final model was obtained by backward elimination, dropping in a stepwise process all of the nonsignificant effects. The statistical tests are the Wald tests for the estimates of the model. To avoid collinearity, scalar variables, fixed and random, were centered, while the categorical variables were sum-coded (i.e. male = 0.5, female = −0.5; u = 0.5, a = −0.5). The results presented here are not affected by collinearity. Table 2 summarizes the parameter estimate 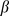 for each of the fixed effects in the model, as well as the estimate of its standard error SE(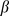), the associated Wald's 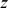-score, and the significance level. As expected, talker voice and vocalic context are both significant predictors of /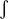/ response. When the talker is male, the odds of a listener hearing /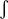/ is approximately a third (0.28) that of a female talker context; the odds of hearing /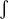/ when the following vowel is /u/ is two-fifths (0.41) that of a following /a/ context. As shown in Figure 1, the effects of vocalic context (left panel) and of talker voice (right panel) on the rate of /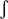/ response also vary depending on the nature of the fricative along the synthesized continuum. Both contextual effects are strongest at the middle range of the fricative continuum when the fricative is most ambiguous. These results are in agreement with previous studies [1], [2]. A significant interaction between talker voice and vocalic context is observed. That is, listeners hear even less /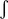/ in the /u/ context when the talker is male than when the talker is female. Of particular interest here is the significant interaction between vocalic context and the overall AQ score. Individuals with high AQ perceptually compensate for the effect the following vowel more than individuals with low AQ. That is, the difference in the rate of context-specific /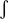/ response attenuates as a function of decreasing overall AQ scores. Figure 2 further illustrates a significant three-way interaction between AQ, vocalic context, and subject's sex, showing that, while females exhibit a decrease in perceptual compensation of vocalic context as a function of decreasing AQ score, no such effect is observed in males. Interaction between talker voice and AQ score was not a significant term. A likelihood ratio test comparing a model with a Talker.Voice x AQ interaction term and one without it shows that the added interaction does not significantly improve model log-likelihood (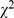 = 2.4753, 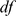 = 1, 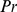
(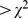) = 0.1157). The overall AQ of an individual is thus not a significant predictor of individual variation in the perceptual compensation for talker voice differences.

**Table 2:** Estimates for all predictors in the analysis of listener response in the identification task.

Predictor	Coef. 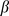	SE( 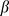 )	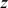	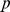
Intercept	−0.6826	0.1877	−3.64	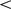 0.001 ***
Trial	−0.0021	0.0017	−1.26	0.2084
Block	−0.0110	0.0884	−0.12	0.9007
Step	0.8653	0.0135	63.99	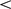 0.001 ***
Talker.Voice	−1.2882	0.0430	−29.93	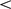 0.001 ***
Vowel	−0.8740	0.0426	−20.51	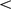 0.001 ***
Subject.Sex	−0.5960	0.3729	−1.60	0.1100
logAQ	0.3917	1.1650	0.34	0.7367
Step×Talker.Voice	−0.1136	0.0230	−4.93	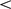 0.001 ***
Step×Vowel	−0.0798	0.0230	−3.46	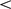 0.001 ***
Talker.Voice×Vowel	−0.6380	0.0827	−7.71	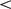 0.001 ***
Vowel×logAQ	−0.6269	0.2593	−2.42	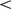 0.05 *
Vowel×Subject.Sex	−0.1305	0.0823	−1.59	0.1129
Subject.Sex×logAQ	0.8076	2.3301	0.35	0.7289
Vowel×Subject.Sex×logAQ	1.0528	0.5187	2.03	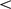 0.05 *

Our regression model shows that AQ is a significant predictor of perceptual compensation for the influence of vocalic context in sibilant classification. Individuals with lower overall AQ scores are less likely to exhibit differences in /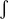/ response as a function of vocalic context. Noteworthy is the fact that this interaction between vocalic context and AQ is sex-differentiated. The AQ effect on vocalic compensation is only evident in women and not in men. Taken together, these results suggest that women with low AQ scores tend to classify as /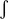/ instances heard by other listeners as /s/ in the /u/ context. From the point of view of sound change, repeated classification discrepancies of this nature might result in the emergence of a sound pattern in the speech of low AQ women where the only sibilant that occur before /u/ is /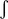/ while /s/ and /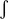/ are both allowed before /a/.

Equally important is the fact that AQ is not a significant predictor for talker voice compensation. Given that failure to compensate for contextual variation in speech is a primary source of novel linguistic variants (new sounds or sound patterns) in a speech community, it is significant that overall AQ scores only affect the compensation for vocalic context and not for talker voice since we know of no sound change or sound pattern that is dependent on talker voice differences; sound patterns that are dependent on vocalic context are plenty, on the other hand.

While our model shows a significant correlation between overall AQ score and compensation for vocalic context, it remains unclear what “autistic” traits might be responsible for reducing vocalic context compensation. A second mixed effects regression model was constructed to examine which of the five subscales of the AQ are significant predictors of /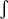/ identification in different vocalic contexts.

### Correlation with AQ subscales

The model contains ten fixed variables: Trial (1–112), Block (1–3), Continuum Step (1–7), Talker.Voice (male vs. female), Vowel (/a/ vs. /u/), Subject.Sex (male vs. female), AQSS(10–40), AQCM (10–40), AQAS (10–40), and AQAD (10–40). The model also includes twelve two-way interactions: Step x Vowel, Step x Talker.Voice, Talker.Voice x Vowel, Vowel x Subject.Sex, Subject.Sex x AQAS, Subject.Sex x AQCM, Vowel x AQAS, Vowel x AQCM, Talker.Voice x AQSS, Talker.Voice x AQAS, Talker.Voice x AQCM, and Talker.Voice x AQAD) and two three-way interactions: Subject.Sex x Vowel x AQAS and Subject.Sex x Vowel x AQCM. Like the previous model, the analysis includes Trial nested within Block as a random factor and two by-subject random slopes, for Trial and for Block. AQAS and AQCM were residualized for the effect of AQSS to avoid collinearity.

**Figure 3:**
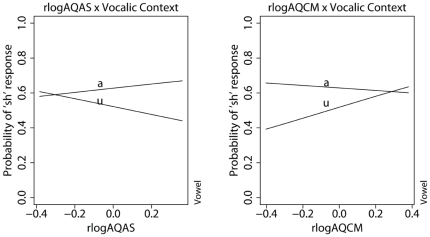
Interaction between vocalic context and subject's log-transformed AQAS and AQCM subscores. The AQAS and AQCM scores were residualized for the effect of AQSS to avoid collinearity.

A summary of the parameter estimates for the fixed effects of the second regression model and their significance is given in Table 3. In addition to the expected two-way interactions already mentioned in the first model, this second model reveals a significant interaction between vocalic context and subject's sex. That is, the odds of men hearing /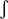/ between the /a/ and /u/ contexts is reduced by a fifth compared to that of the women, suggesting that men are, in general, more robustly compensating for vocalic context perceptually than women. Turning to the effects of subscale scores on perceptual compensation, the model reveals that vocalic context compensation is influenced by the listener's ability to switch attention (residualized AQAS) and to communicate (residualized AQCM), modulo the effect of social skills (AQSS) on these abilities. Figure 3 show that, when a listener has high residualized AQAS, which may indicate poor attention-switching and over-fixation in attention, s/he is also better at compensating for vocalic context. On the other hand, individuals who have low residualized AQCM, which may indicate good communication skills, are better at compensating for vocalic context than those with high residualized AQCM.

**Table 3:** Estimates for all predictors in the analysis of listener response in the identification task.

Predictor	Coef. 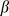	SE( 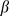 )	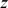	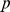
Intercept	−0.6230	0.1887	−3.30	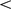 0.001 ***
Trial	−0.0021	0.0017	−1.26	0.2083
Block	−0.0111	0.0897	−0.12	0.9016
Step	0.8738	0.0137	63.97	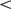 0.001 ***
Talker.Voice	−1.3021	0.0434	−30.04	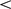 0.001 ***
Vowel	−0.9349	0.0439	−21.31	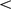 0.001 ***
Subject.Sex	−0.6534	0.3769	−1.73	0.0829
rlogAQAS	0.2017	1.2573	0.16	0.8725
rlogAQCM	0.4438	1.3268	0.33	0.7380
logAQSS	−0.2677	0.6598	−0.41	0.6849
logAQAD	0.9753	0.9328	1.05	0.2958
Step×Talker.Voice	−0.1083	0.0232	−4.68	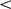 0.001 ***
Step×Vowel	−0.0855	0.0233	−3.67	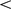 0.001 ***
Talker.Voice×Vowel	−0.6423	0.0832	−7.72	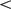 0.001 ***
Vowel×Subject.Sex	−0.2179	0.0850	−2.56	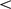 0.05 *
Subject.Sex×rlogAQAS	−2.3766	2.6188	−0.91	0.3641
Subject.Sex×rlogAQCM	−2.1684	2.6174	−0.83	0.4074
Vowel×rlogAQAS	−2.0677	0.2829	−7.31	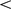 0.001 ***
Vowel×rlogAQCM	2.0220	0.3004	6.73	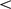 0.001 ***
Talker.Voice×logAQSS	−0.5248	0.1407	−3.73	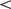 0.001 ***
Talker.Voice×rlogAQAS	−0.6550	0.2659	−2.46	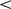 0.05 *
Talker.Voice×logAQAD	0.5459	0.2122	2.57	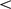 0.05 *
Talker.Voice×rlogAQCM	0.8480	0.2512	3.38	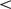 0.001 ***
Vowel×Subject.Sex×rlogAQAS	2.5414	0.5647	4.50	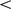 0.001 ***
Vowel×Subject.Sex×rlogAQCM	2.2316	0.6000	3.72	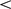 0.001 ***

To eliminate collinearity, in addition to the canonical centering and sum-coding, logAQAS and logAQCM were residualized for the effect of logAQSS.

**Figure 4:**
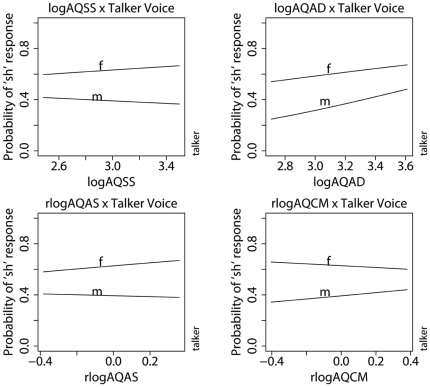
Interaction between talker voice and subject's log-transformed AQSS, AQAD, AQAS and AQCM subscores. The AQAS and AQCM scores were residualized for the effect of AQSS.

The nature of the subscore's influence on vocalic context normalization varies as a function of the subject's sex. For each unit of rAQAS, the odds of male listeners being influenced by the effect of attention switching (rAQAS) on vocalic context compensation is half (0.49) that of the female listeners. Likewise, for each unit of rAQCM, the odds of men being influenced by the effect of communication skills on vocalic context compensation is almost half (0.45) that of the women's. Turning now to the effects of AQ subscores on the normalization for talker voice, as illustrated in Figure 4, four of the five subscale scores are significant predictors of talker voice compensation. The model shows that a listener with poorer social skills (high AQSS) and low attention-switching abilities (high residualized AQAS) is better at compensating for talker voice differences. On the other hand, individuals who have poor communication skills (low residualized AQCM) and abilities to attend to details (low AQAD) are better at talker voice compensation. An important difference between talker voice and vocalic context compensation is that they are affected by the listener's cognitive processing style in different ways. A quick comparison between Figures 3 and 4 and the coefficients of the interaction terms shows that the effects of AQ subscale scores on talker voice compensation are much weaker than their effects on vocalic compensation. The coefficients of the interactions between vocalic context and the residualized AQAS and AQCM subscores are almost three times larger than the coefficients of the interactions between the same residualized subscores and talker voice. This might explain why the overall AQ score does not have an effect on talker voice compensation in general.

## Discussion

The present study shows that the magnitude of perceptual compensation varies as a function of cognitive processing style, as measured by individuals' overall and subscale AQ scores. The effects of cognitive processing style vary depending on the type of contextual information in question as well as on the sex of the listener. With respect to the influence of vocalic context, women in general, and especially those with low overall AQ, show a reduction in perceptual compensation compared to their male counterparts. Individuals with good attention-switching abilities and poor communication skills are also prone to under-compensate for vocalic context effects. With respect to talker voice differences, individuals with good social skills and attention-switching abilities show a decrease in normalization for talker voice differences in speech. On the other hand, individuals with poor communication skills and abilities to attend to detail and patterns are also less likely to compensate for talker voice effects. Despite the AQ subscale effects on talker voice compensation, it is noteworthy that such subscale effects are much smaller than the AQ subscale effects on vocalic context compensation, as evidenced by the facts that the regression coefficients for the talker voice interaction with AQ subscale scores are less than half that of the vocalic context interaction with AQ subscale scores. This is consistent with the observation that sound changes conditioned by talker voice are virtually unheard of, but sound changes conditioned by vocalic contexts are plenty. The fact that the Attention-Switching and Communication subscales of AQ are significant predictors of both types of perceptual compensatory responses (i.e. vocalic context and talker voice) strongly suggests that perceptual compensation is partly governed by social interactional factors [50]. Difficulties in attention switching, communication, and, to a smaller extent, social skills make it harder to keep track of social information. While this marked deficit in attention switching and communication in ASC subjects and neurotypicals with high degree of “autistic” traits may directly harm their social and communication abilities, it also seems to have allowed them, at least the high AQ neurotypicals, to focus their attention on lower level contextual cues, since keeping track of social information usually involves frequent and rapid changes in the source of information (visual or auditory information, change in objects or actions, etc.), and requires the ability to follow the flow of social cues (words, gestures, postures, background context, etc) [51]. This tradeoff in cognitive resource allocation might explain why high AQ individuals are better able to take vocalic context and talker voice into account than individuals who are more distracted by the multi-dimensional nature of social interaction. The fact that superior abilities to attend to detail (high AQAD) correlates negatively with talker voice compensation, on the other hand, suggests that, despite the superior perceptual functioning as hypothesized by the EPF model, the more robust memory trace of contextual variants of speech categories might actually hamper high AQ individuals' abilities to compensate for contextual variation. Such individuals might rely more on the surface properties of the auditory patterns, rather than take into account the contextual cues in categorizing the speech signal. Taken together, the results of this study are consistent with the hypothesis that individuals with holistic cognitive processing style are focusing more on recovering the higher-level meaning of the message at the expense of attending to context-induced fluctuations at the level of the individual speech sounds.

Returning to the issue of how differences in cognitive processing style might affect sound change, recall that a main source of variation in language has been hypothesized to be the consequence of listeners failing to normalize for the effects of context on the realization of speech sounds. The present findings suggest that there exists a subsection of a speech community (i.e. women with low AQ) that are regularly under-compensating and misparsing (e.g., a /su/ sequence might be interpreted as /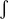
u/, but /sa/ is heard correctly as /sa/). Such misperception does not necessarily lead to miscommunication 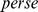 given the highly redundant nature of speech [52]; the listener can accurately identify the meaning of a message even if s/he fails to correctly identify the individual speech sounds that encode the message. Given the systematicity of misparsing, individuals who consistently under-compensate for contextual effects in speech are likely to have different perceptual and pronunciation norms than individuals who succeed in perceptual compensation, assuming that perceptual experience informs articulatory production. In this vein, it is noteworthy that this propensity to under-compensate is gender-differentiated. Females are more likely to under-compensate than males (regardless of scores, as revealed in the second regression model) and females with lower AQ under-compensate more than females with higher AQ. Studies in social dialect variation and gender have repeatedly observed that women make use of a wider range of variation than men [11], [12], [53], [54] and females are often the more active agents of the diffusion of sound change compared to men (see [12], [55]; cf. [56]). Some attribute women's greater use of linguistic resources than men to their reliance on symbolic expressions and means to attain worth and power (especially economic power) [54]. The present study suggests that a contributing factor to women having access to a wider range of linguistic resources (i.e. linguistic variation) might be biologically-based; women's superior ability to retain variants in speech than men might be the result of their propensity to under-normalize for contextual variation (see [57] and [58] for other proposals of a biologically-based cognitive foundation of gender-differentiation in linguistic variation and change). The observed differences within the sexes is also significant in that it highlights the non-monolithic nature of gender categories, mirroring findings of decades of sociolinguistic research on gendered variation in speech. Finally, research on the broader phenotypes of ASC has found that high AQ individuals are associated with high neuroticism, low extraversion, and low agreeableness [45] or conscientiousness [46]. Taken together, the portion of the neurotypical population that is the least able to compensate for context-induced variation might also have the type of personality traits that would excel in social interaction (i.e. highly extroverted, more agreeable and conscientious), and such individuals might also have the type of social profiles that facilitate innovation and leadership in linguistic change within a speech community. To be sure, the eco-sociological influence of the observed cognitive processing style variation in shaping the dynamics of language change requires further empirical investigation. The present study offers at least suggestive clues for the cognitive, and possibly neurobiological, foundation of socially-motivated linguistic change.

## Materials and Methods

### Participants

Sixty university students (32 females), all native speakers of American English, took part in the study either for course credit or for a nominal fee. Their age range from 18–47, with a mean of 22 (SD = 4.7). All participants took the /s/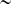/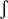/ identification task and filled out the AQ questionnaire.

### Stimuli

Four /sV-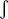
V/ continua were created (V = /a/ or /u/). The fricative portion of the continuum was created by digitally mixing in 5% increments various mixtures (a weighted average of the waveforms) of the /s/ and /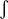/ sounds taken from clear tokens of /sa/ and /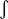
a/ produced by a female native speaker of American English (e.g., 5% /s/ mixed with 95% /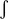, 10% /s/ mixed with 90% /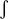/, etc). Five steps were selected: 65% /s/ mixed with 35% /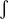/, 50% /s/+50% /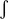/, 35% /s/+65% /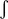/, 20% /s/+80% /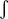/, and 10% /s/+90% /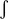/. The original /s/ and /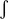/ were included as endpoints of the seven-step series. The seven fricatives (synthesized and natural) were then cross-spliced with /a/ and /u/ taken from original /da/ and /du/ syllables produced by two native speakers of American English, one male and one female. The resulting tokens were then normalized for intensity and pitch. The final stimuli were judged by two native speakers of English to be natural sounding.

### Procedure

Subjects categorized the /sV-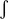
V/ continua by identifying each initial sibilant as either /s/ or /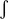/. The experiment was implemented in E-Prime. Subjects heard the test stimuli over headphones in a sound-proof booth. Subjects made their selection by pressing one of two labeled keys on a response box. The session consisted of three trial blocks. In each block, all 28 tokens ( = 2 vowels×2 talkers×7 steps) were presented four times in random order. Each subject categorized 336 tokens ( = 2 vowels×2 talkers×7 steps×3 blocks×4 times). After the identification task, participants took the Autism-Spectrum Quotient questionnaire (AQ: [38]), which is a short, self-administered scale for identifying the degree to which any individual adult of normal IQ may have traits associated with ASC. The AQ is not a diagnostic measure, although it has been clinically tested as a screening tool; traits as assessed by the AQ show high heritability and are stable cross-culturally. The test consists of 50 items, made up of 10 questions assessing five subscales: social skills (SS), communication (CM), attention to detail (AD), attention-switching (AS), and imagination (IM). The test was administered as a pen-and-paper task. Participants answered the question possible by circling their response on a 4-point scale (‘strongly disagree’, ‘disagree’, ‘agree’, and ‘strongly agree’).

### Scoring

The AQ items were scored on a Likert scale (1–4). A total AQ score was calculated by summing all of the scores for each of the items, with a maximum score of 200 and a minimum score of 50. Scores for the subscales (AQSS, AQCM, AQAD, AQAS, AQIM) have a maximum score of 40 and a minimum score of 10. All scales were scored in such a way that a high score is more “autistic”, i.e. lower social skills, difficulty in attention switching/strong focus of attention, high attention to detail and patterns, lower ability to communicate, and low imagination. Overall and subscale AQ scores were log-transformed in the analysis.

### Ethics Statement

The study was approved by the Social and Behavioral Sciences Institutional Review Board at the University of Chicago and verbal informed consent was obtained from all participants.
